# *CYP4F12* is a potential biomarker and inhibits cell migration of head and neck squamous cell carcinoma via EMT pathway

**DOI:** 10.1038/s41598-023-37950-z

**Published:** 2023-07-06

**Authors:** Wenming Jia, Shuai Chen, Ran Wei, Xiaoqi Yang, Minfa Zhang, Ye Qian, Heng Liu, Dapeng Lei

**Affiliations:** 1 Department of Otorhinolaryngology, Qilu Hospital of Shandong University, National Health Commission (NHC) Key Laboratory of Otorhinolaryngology (Shandong University), Jinan, China; 2 Department of Otolaryngology/Head and Neck Surgery, Institute of Otolaryngology, Affiliated Hospital of Binzhou Medical University, Binzhou, China

**Keywords:** Head and neck cancer, Metastasis, Tumour biomarkers

## Abstract

Head and neck squamous cell carcinoma (HNSC) is the most common malignant tumor of head and neck. Due to the insidious nature of HNSC and the lack of effective early diagnostic indicators, the development of novel biomarkers to improve patient prognosis is particularly urgent. In this study, we explored and validated the correlation between cytochrome P450 family 4 subfamily F member 12 (CYP4F12) expression levels and HNSC progression using data from The Cancer Genome Atlas (TCGA), Gene Expression Omnibus (GEO) datasets and collected patient samples. We analyzed the association of CYP4F12 expression with clinicopathological features, immune correlation and prognosis. Finally, we analyzed the correlation between CYP4F12 and pathways, and verified by experiments. The results showed that CYP4F12 was low expressed in tumor tissues, participated in a variety of phenotypic changes of HNSC and affected immune cell infiltration. Pathway analysis indicated that CYP4F12 may play a key role in tumor cell migration and apoptosis. Experimental results showed that over-expression of CYP4F12 inhibited cell migration and enhanced the adhesion between cells and matrix by inhibiting epithelial-mesenchymal transition (EMT) pathway in HNSC cells. In conclusion, our study provided insights into the role of CYP4F12 in HNSC and revealed that CYP4F12 may be a potential therapeutic target for HNSC.

## Introduction

HNSC, which accounts for 90% of all head and neck cancers, is the sixth most common type of cancer in the world^1,2^. HNSC develops in the mucosa membranes of the nasal, oral, hypopharyngeal and laryngeal cavities^3,4^. HNSC is usually associated with exposure to tobacco-derived carcinogens, excessive alcohol consumption and human papillomavirus (HPV) infection^5,6^. Although treatments have improved over the past forty years, overall survival rates have not changed significantly^7,8^. Therefore, it is crucial to further investigate the pathogenesis of HNSC and develop predictive biomarkers to improve the detection and survival of patients with HNSC.

Human cytochrome P450 (CYP450) superfamily is a polygenic family of enzymes consisting of 18 families that are expressed in many tissues^9,10^. CYP450 enzymes are involved in the oxidative metabolism of a variety of endogenous compounds (such as lipids and steroids) and exogenous compounds (such as drugs and toxins)^11,12^. Recently, the literature has shown that these enzymes may also play an important role in tumorigenesis^13–15^. They can directly participate in tumor initiation and progression^16,17^, or participate in the process of tumors through some metabolites^18–20^, or affect tumor treatment by metabolizing chemotherapeutic drugs^21,22^. A growing body of literature suggests that members of the CYP450 family are critically involved in cancer progression. However, the role and mechanisms of CYP4F12 in HNSC remain unclear.

In this study, we examed the expression of CYP4F12 in a variety of tumor types, explored its latent role in HNSC immune infiltration, and evaluated its prognostic value in HNSC patients. In addition, we investigated the molecular alterations and immune signatures of HNSCs and assessed their impact on clinical outcomes. We further analyzed the differentially expressed genes and functional enrichment associated with CYP4F12 expression. Finally, we performed in vitro experiments to determine the inhibitory effects of CYP4F12 on HNSC cell migration and adhesion and explored the potential underlying mechanism. The aim of the current study was to confirm whether CYP4F12 could be a promising predictive biomarker for the prognosis of HNSC and the response to immunotherapy.

## Materials and methods

### Access to public datasets

The expression of CYP4F12 in a variety of cancer cell lines and specific HNSC cell lines was analyzed using the Cancer Cell Line Encyclopedia (CCLE) data set (https://portals.broadinstitute.org/ccle/about) with the R V4.0.3 software (https://www.r-project.org/) package GGplot2 (v3.3.3)^23^.

A pan-cancer analysis of CYP4F12 was performed using TIMER, a database capable of exploring gene expression across different tumor types^24^ (https://cistrome.shinyapps.io/timer).

The expression of CYP4F12 in HNSCs and adjacent non-tumor tissues was investigated using TCGA HNSC dataset, as well as the GSE107591 (23 normal and 24 tumor tissues) and GSE58911 (15 paired normal and tumor samples) datasets retrieved from the GEO database (https://www.ncbi.nlm.nih.gov/geo), which were analyzed using the GEO2R online program. A total of 543 HNSC cases were retrieved from TCGA HNSC. Raw count data were provided by the submitter.

Potential correlations between CYP4F12 expression and clinicopathologic variables of HNSC patients were determined using the Ualcan database (http://ualcan.path.uab.edu/), a website for analyzing cancer omics data that provides comprehensive transcriptomic data for cancers from TCGA^25,26^.

Overall survival (OS) and disease-free survival (DFS) of CYP4F12 were analyzed using the gene expression profiling interactive analysis (GEPIA) database platform (http://gepia.cancer-pku.cn), an online tool for systematic analysis of gene expression^27^.

In addition, the RNA-seq data (level 3) and corresponding clinical information of 504 HNSC patients were obtained from TCGA database (https://portal.gdc.com). TimeROC (v 0.4)^28^ was used to analyze the receiving operating characteristics (ROC) curves and area under ROC curves (AUCs) of CYP4F12. The predictive potential of CYP4F12 was assessed. Patients were divided into two groups according to the median of CYP4F12 transcripts, and the R software packages ggstatsplot (v0.11.1)^29^ and pheatmap (v1.0.12)^30^ were used for immune correlation and immune cell biomarker analysis, respectively. Differences in immune scores, immune checkpoints, immune checkpoint blockade (ICB) response analysis, and clinical data between the two groups were also analyzed. The R package immunedeconv, which integrates six algorithms, was used to evaluate the immune score, and the TIMER algorithm was used to calculate the immune score between groups. Immune checkpoints were calculated by extracting the expression gene values of SIGLEC15, TIGIT, CD274, HAVCR2, PDCD1, CTLA4, LAG3 and PDCD1LG2 to observe the correlation between the expression of immune checkpoint-related genes and CYP4F12. These results were obtained using the R packages GGplot2 and pheatmap. For ICB response analysis, the Tumor Immunodeficiency and Exclusion (TIDE) algorithm was used to predict potential immunotherapy responses.

Differentially expressed gene analysis was performed using the R software package Limma (v3.40.6)^30^. Adjusted *P* < 0.05 and log2 (fold change) > 1 or log2 (fold change) < − 1″ were defined as thresholds for mRNA differential expression screening. Data were analyzed by feature enrichment to confirm the potential functions of potential targets, using ClusterProfiler (v4.2.2) package^31^ in R to analyze the Gene Ontology (GO) function of potential mRNAs and enrich the Kyoto Encyclopedia of Genes and Genomes (KEGG) pathway^31^.

To analyze the correlation between CYP4F12 and HNSC pathway, we collected the set of genes included in the relevant pathway^32^, analyzed by the R software GSVA (v1.40.1) package^33^, selected the parameter method = 'ssgsea', the correlation between genes and pathway scores was analyzed by Spearman correlation.

### Patients and tumor samples

Clinically resected tumor specimens were randomly collected from 22 patients with HNSC cancer who underwent curative resection with informed consent at Qilu Hospital of Shandong University, between 2019 and 2021. These patients did not receive any local or systemic chemotherapy before their surgery. All samples were collected and immediately frozen in liquid nitrogen and stored at − 80 °C. Quantitative real-time polymerase chain reaction (qRT-PCR) was used to determine CYP4F12 gene expression. This study was approved by the Ethics Committee of Qilu Hospital of Shandong University (approval number: KYLL-2019-2-095; expiration date: 2019–2021).

### Cell culture

The human HNSC cell lines FaDu (human hypopharynx cancer cells, ATCC HTB-43) and SCC-9 (human oral cavity cancer cells, ATCC CRL-1629) were purchased from the ATCC. All media were added with 10% fetal bovine serum (FBS) (Minhai Bio-engineering Co., LTD) and 1% penicillin–streptomycin solution (Beyotime). All cell lines were cultured within 2 months of resuscitation under standard conditions at 37 °C, 5% CO_2_. FaDu cell line was cultured in MEM, and SCC-9 cell line was cultured in DMEM.

### Plasmid, siRNA and cell transfection

Plasmid was purchased from Weizhen Biology (China). Double-stranded siRNAs against human CYP4F12 (siRNA1 targeting sequence: GAAGCCAGCAUAUCCUCCATT; siRNA2 targeting sequence: GAAGCCAGCATATCCTCCATT) and control siRNA was purchased from GeneChem (China). According to the manufacturer's protocol, CYP4F12 was transfected using Lipofectamine 3000 (Thermo Fisher). FaDu cells (1.6 × 10^5^ cells/mL; 2 mL), SCC-9 cells (1.1 × 10^5^ /mL; 2 mL) were seeded in 6-well plates and cultured for 24 h. Cells were then transfected with empty vector plasmid and CYP4F12 plasmid, or CYP4F12 siRNA1, siRNA2 and control siRNA. Cells were harvested after 48 h for subsequent experiments.

### Cell viability

Approximately 5 × 10^3^ transiently transfected FaDu cells were seeded into 96-well plates. After incubation for 24, 48, and 72 h, 10 µL of Cell Counting Kit-8 (CCK-8) solution was added to each well. After 2 h of incubation, the optical density (OD) was detected at 450 nm using a microplate reader (Bio-Rad, USA).

### Cell cycle and apoptosis analysis

Cell cycle was detected using the Cell Cycle Assay Kit (Bestbio). Approximately 2 × 10^5^ transiently transfected FaDu cells were seeded in 6-well plates for 48 h. Cells were treated with trypsin and collected, fixed with 75% cold ethanol, and incubated at − 20 °C for 1 h. After incubation, 20 μ L RNase was added to the cell suspension and incubated at 37 °C for 30 min. Cells were centrifuged, and the supernatant was resuspended with 0.5 mL of PI staining buffer and incubated at room temperature in the dark for 15 min. Results were quantified using CytEpert V2.0 (Beckman Coulter).

Annexin V-FITC/PI apoptosis assay kit (Bestbio) was used to detect apoptosis. Approximately 2 × 10^5^ transiently transfected FaDu cells were seeded in 6-well plates for 48 h. Cells were collected with EDTA-free trypsin. Cells were then resuspended in binding buffer and double stained with fluorescein isothiocyanate (FITC) and propidium iodide (PI). Apoptotic cells were immediately measured by flow cytometry (Beckman Coulter, Cytoflex S). The whole procedure was performed in the dark.

### Transwell assay

Migration assays were performed in Transwell chambers with 8 µm pore size filters (Costar). The upper compartment was plated with 5 × 10^4^ cells in serum-free medium, while 20% FBS was added to the lower compartment. The cells were cultured for 24 h, then washed with phosphate buffered saline (PBS) and fixed with methanol. The cells were stained with 0.1% crystal violet stain (Solarbio). Finally, the stained cells were photographed and counted.

### Wound healing assay

Approximately 5 × 10^5^ transiently transfected cells were inoculated into 6-well plates and cultured for 24 h to 80% to 90% confluence. A 200 µl sterile pipet tip was scraped in the center of each well to create a scratch. After washing with PBS, MEM or DMEM medium with 1% FBS was added to the plate. Scratches were photographed every 12 h. Cell migration rate was quantified by comparing the images.

### Cell-matrix adhesion assay

The cell adhesion assay kit (BestBio) was used to analyze the adhesion capacity of the CYP4F12 function in FaDu and SCC-9 cells. The coating buffer (100 µL/ well) was added to 96-well cell plates, and incubated overnight at 4 °C. The next day, the coating buffer was removed and washed 3 times with the washing solution. Then, cells were harvested with trypsin 48 h after transfection, washed with PBS and resuspended in MEM or DMEM medium. Then, 1 × 10^5^ cell were seeded into each pretreated 96-well plate. 10 multiple wells were set up for each sample, 5 for the experimental group and 5 for the control group. Cells were incubated at 37 °C for 30 min. Next, the control group was left untreated, and the experimental group was washed 3 times with 100 µL of culture medium. Then 10 µL of staining solution B was added, and incubated at 37 °C for 1 h. The optical density (OD) was measured at 450 nm using a microplate reader (Bio-Rad, USA). The adhesion rate of each group was calculated as (OD-CYP4F12 − OD-CYP4F12-blank) / (OD-Control − OD-Control-blank) * 100%.

### Western blot

Cells were lysed using an ultrasonic cracker in RIPA buffer (Thermo Fisher) with protease inhibitor (Sigma). Protein concentrations were measured using the BCA Protein Assay Kit (Beyotime). After electrophoresis, proteins were transferred to polyvinylidene fluoride (PVDF) membranes. The PVDF membrane was sealed and incubated with primary and secondary antibodies. The bands were scanned using an infrared fluorescence scanning imager, and the gray values of the protein bands were analyzed using ImageJ software. The following antibodies were used: CYP4F12 antibody (Proteintech, 13243-1-AP), E‐cadherin (Abcam, ab40772), N‐cadherin (Abcam, ab76011), vimentin (Abcam, ab92547), α‐catenin (Abcam, ab51032), anti-mouse IgG (H + L) (DyLight™ 680 4X PEG Conjugate) (Cell Signaling Technology 5470S), anti-rabbit IgG (H + L) (DyLight™ 800 4X PEG Conjugate) (Cell Signaling Technology 5151S), and β‐actin (Abcam, ab8226) antibody.

### Statistical analysis

Statistical analysis was performed using the above online databases and R-package. Correlations between CYP4F12 expression and other genes were calculated and evaluated using Spearman correlation coefficients. Student's t-tests were used for continuous variables. Pearson's chi-square test and Fisher's exact test were used to compare categorical variables. All *p*-values tested and reported are two-tailed, different levels of significance were used: *p-value < 0.05; **p-value < 0.01; and ***p-value < 0.001 were considered statistically significant.

## Results

### Expression of CYP4F12 in different cancers and cultured cancer cells

**Figure 1:**
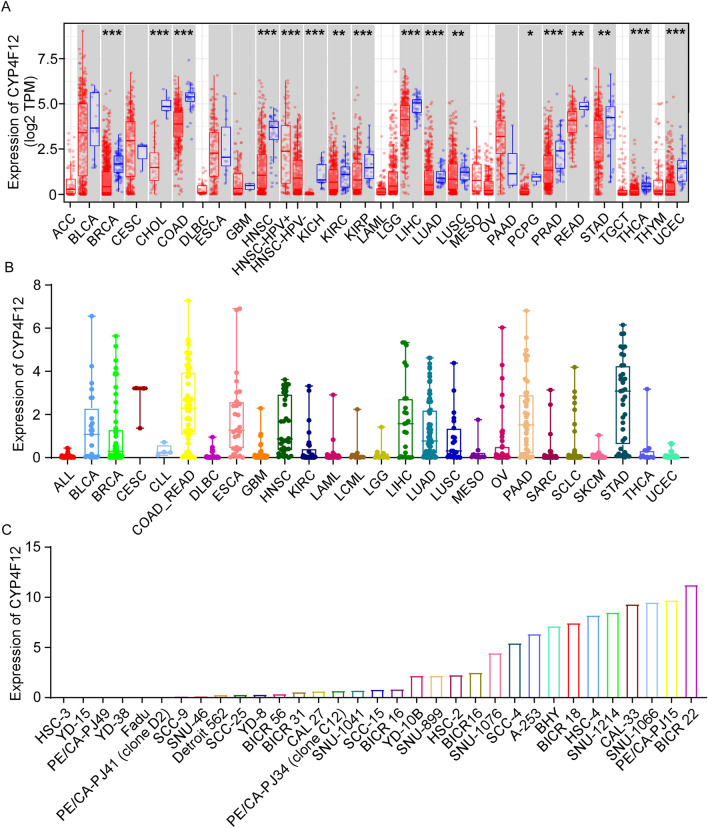
Expression levels of CYP4F12 gene in different cancers and cultured cancer cells. (**A**) CYP4F12 expression in pan-cancer analysis using TIMER. (**B**) CYP4F12 expression in different cell lines from CCLE. The ordinate is the expression of CYP4F12, while the abscissa designates cell lines categorized by their origin. (**C**) Expression of CYP4F12 in different HNSC cell lines from CCLE. (*p < 0.05, **p < 0.01, ***p < 0.001).

To clarify the importance of CYP4F12 expression in tumors, we first analyzed CYP4F12 expression in a variety of cancers using TIMER. As shown in Fig. 1A, the expression of CYP4F12 mRNA was lower in almost all tumor tissues than in their matched, especially in breast invasive carcinoma (BRCA), cholangiocarcinoma (CHOL), colon adenocarcinoma (COAD), HNSC, kidney chromophobe (KICH), kidney renal papillary cell carcinoma (KIRP), liver hepatocellular carcinoma (LIHC), lung adenocarcinoma (LUAD), prostate adenocarcinoma (PRAD), thyroid carcinoma (THCA) and uterine corpus endometrial carcinoma (UCEC). In addition, the expression level of CYP4F12 was significantly higher in HNSC patients with HPV infection than in those without. We then determined the expression of CYP4F12 in various cultured tumor cells based on the RNA-SEQ data obtained from the CCLE database. The result (Fig. 1B) showed that CYP4F12 is expressed in all tumor cell lines, but generally at low levels. Mean CYP4F12 expression levels of cell lines were higher in bladder urothelial carcinoma(BLCA), colon adenocarcinoma and rectum adenocarcinoma (COAD_READ), HNSC, LIHC, LUAD, pancreatic adenocarcinoma (PAAD) and stomach adenocarcinoma (STAD), while nearly undetectable in those from acute lymphoblastic leukemia (ALL), lymphoid neoplasm diffuse large B-cell lymphoma (DLBC), glioblastoma multiforme (GBM), acute myeloid leukemia (LAML), brain lower grade glioma (LGG), mesothelioma (MESO), sarcoma (SARC), small cell lung cancer (SCLC), skin cutaneous melanoma (SKCM), THCA and UCEC. Next, we examined the transcription level of CYP4F12 in different HNSC cell lines collected in the CCLE database. The results (Fig. 1C) showed that the abundance of CYP4F12 was most prominent in cells such as BICR22, PE/CA-PJ15, SNU-1066 and CAL-33, while almost neglectable in cells such as HSC-3, YD-15, PE/CA-PJ49, YD18, FaDu, PE/CA-PJ41 and SCC-9.

**Figure 2:**
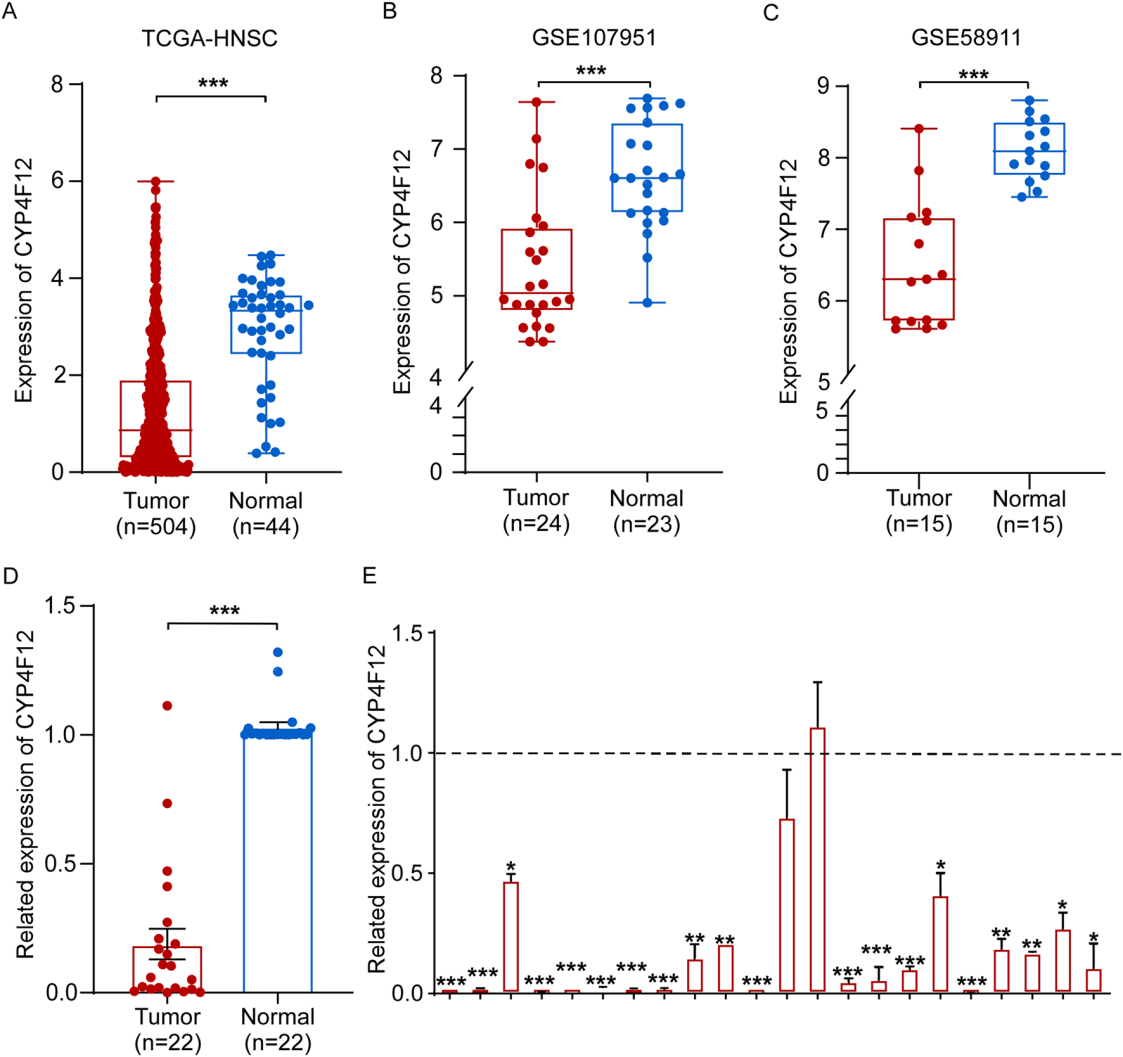
CYP4F12 expression in HNSC. (**A**) CYP4F12 expression in HNSC tumors and normal tissues from TCGA. (**B** and **C**) CYP4F12 expression in HNSCs in the microarray data of GSE107951 and GSE58911 from the GEO database. (**D** and **E**) Relative expression levels of CYP4F12 in 22 pairs of HNSC tissues and adjacent normal tissues. (*p < 0.05, **p < 0.01, ***p < 0.001).

We then sought to confirm the down-regulation of CYP4F12 in HNSCs. Using datasets from TCGA, GSE107951 and GSE58911, we found that CYP4F12 was significantly downregulated in HNSCs compared to adjacent nontumor tissues (Fig. 2A–C). This was further validated in 22 pairs of HNSCs and adjacent non-tumor tissues collected at our institution. (Fig. 2D, E).

### Relationship between CYP4F12 expression and the clinicopathological features of HNSC

**Figure 3:**
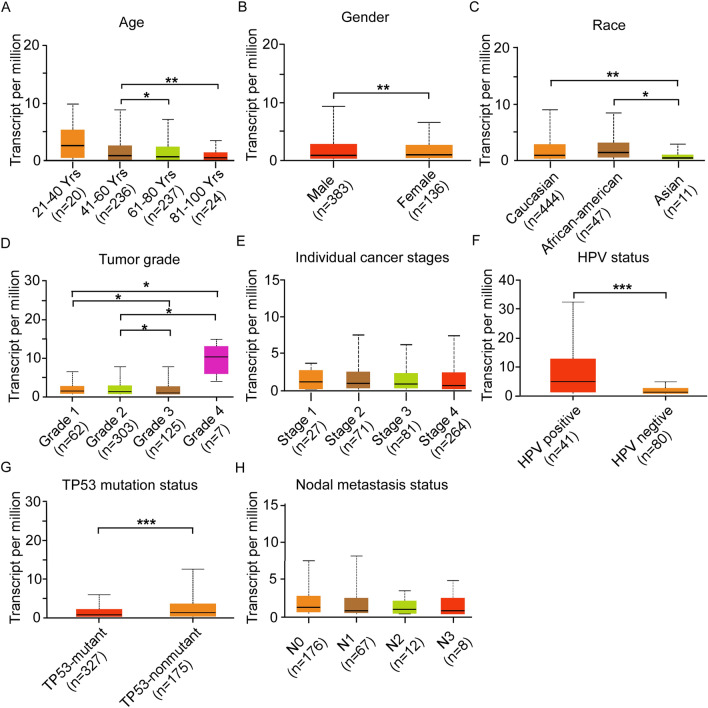
Relationship between CYP4F12 expression and clinicopathological features of HNSC. The expression of CYP4F12 was analyzed between/among different age groups (**A**), sexes (**B**), races (**C**), tumor grades (**D**), cancer stages (**E**), HPV statuses (**F**), TP53 mutation statuses (**G**), and nodal metastasis statuses (**H**). (*p < 0.05, **p < 0.01, ***p < 0.001).

To further explore the clinical implication of CYP4F12 in HNSC, we first classified patients in the TCGA HNSC dataset with different clinicopathologic characteristics, including age, gender, race, tumor grade, individual cancer stage, HPV status, TP53 mutation status and nodal metastasis status. We then analyzed the expression pattern of CYP4F12 within each category (Fig. 3). The date showed that the expression level of CYP4F12 gradually decreased with age. Furthermore, the expression level of CYP4F12 was lower in males, Asian population, HPV-negative patients and TP53 mutant patients than in patients in the other groups. In addition, the expression of CYP4F12 gradually decreased with the increase of tumor grade from grade 1 to grade 3, while peaked at grade 4. However, there was no significant change in CYP4F12 expression level among different tumor stages or lymph node metastasis status. These results suggest that CYP4F12 may be involved in a variety of HNSC phenotypic changes and may play an important role in HNSC.

### The relationship between CYP4F12 and immune infiltration

**Figure 4:**
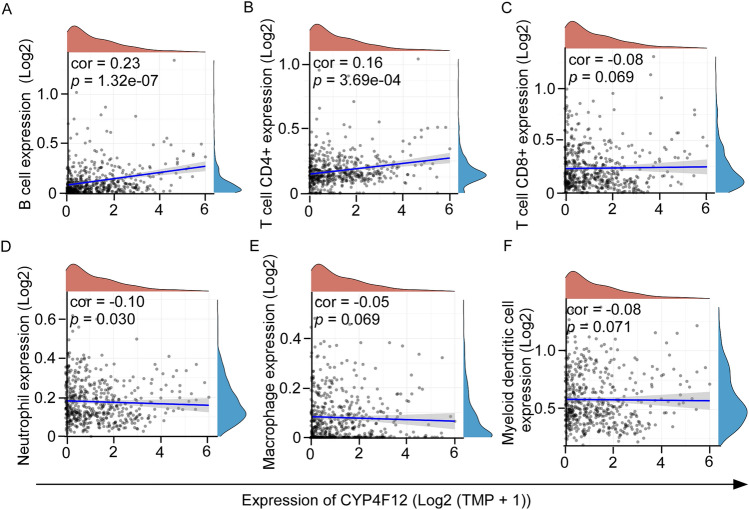
Spearman correlation analysis between CYP4F12 expression and immune score. Spearman correlation analysis between CYP4F12 expression with the expression of B cells (**A**), CD4 + T cells (**B**), CD8 + T cells (**C**), Neutrophil (**D**), Macrophage (**E**) and Myeloid dendritic cells (**F**). The horizontal coordinate indicates CYP4F12 expression and the vertical coordinate indicates the immune score. The density curve on the right side shows the distribution trend of the immune score, and the density curve on the top shows the distribution trend of CYP4F12 expression. The upper values indicate the correlation p-values and correlation coefficients.

Next, we investigated the relationship between CYP4F12 and immune infiltration in HNSC. We found that the expression level of CYP4F12 was positively correlated with the expression of B cells (Fig. 4A) and CD4+ T cells (Fig. 4B), but was not significantly correlated with the expression of CD8+ T cells (Fig. 4C), neutrophils (Fig. 4D), macrophages (Fig. 4E) and myeloid dendritic cells (Fig. 4F).

To further clarify the role of CYP4F12 in tumor immunity, a correlation analysis between CYP4F12 expression and HNSC immune cell markers was performed using the TCGA HNSC dataset. As shown in Table 1, CYP4F12 expression level was significantly and positively correlated with immune cell markers, including B cell markers (CD19 and CD79A), CD8+ T cell markers (CD8B), M1 macrophage markers (NOS2 and IRF5), neutrophil markers (CEACAM8, ITGAM and CCR7) and dendritic cell markers (CD1C). These results suggest that CYP4F12 may influence immune cell infiltration in HNSC.

**Table 1:** Correlation analysis of CYP4F12 and immune cell biomarkers based on the TCGA HNSC dataset.

Immune cell	Biomarker	Correlation	*p*-value
B Cell	CD19	0.281	< 0.001
	CD79A	0.278	< 0.001
CD8+ T Cell	CD8A	0.066	0.138
	CD8B	0.109	0.014
CD4+ T Cell	CD4	0.038	0.397
M1 macrophage	NOS2	0.288	< 0.001
	IRF5	0.203	< 0.001
	PTGS2	0.023	0.602
M2 macrophage	CD163	− 0.164	< 0.001
	VSIG4	− 0.169	< 0.001
	MS4A4A	− 0.160	< 0.001
Neutrophil	CEACAM8	0.184	< 0.001
	ITGAM	0.111	0.012
	CCR7	0.179	< 0.001
Dendritic Cell	HLA-DPB1	0.009	0.843
	HLA-DQB1	− 0.035	0.431
	HLA-DRA	0.000	0.996
	HLA-DPA1	0.005	0.906
	CD1C	0.222	< 0.001
	NRP1	− 0.283	< 0.001
	ITGAX	0.002	0.971

### High expression of CYP4F12 is associated with better prognosis in HNSC patients

**Figure 5:**
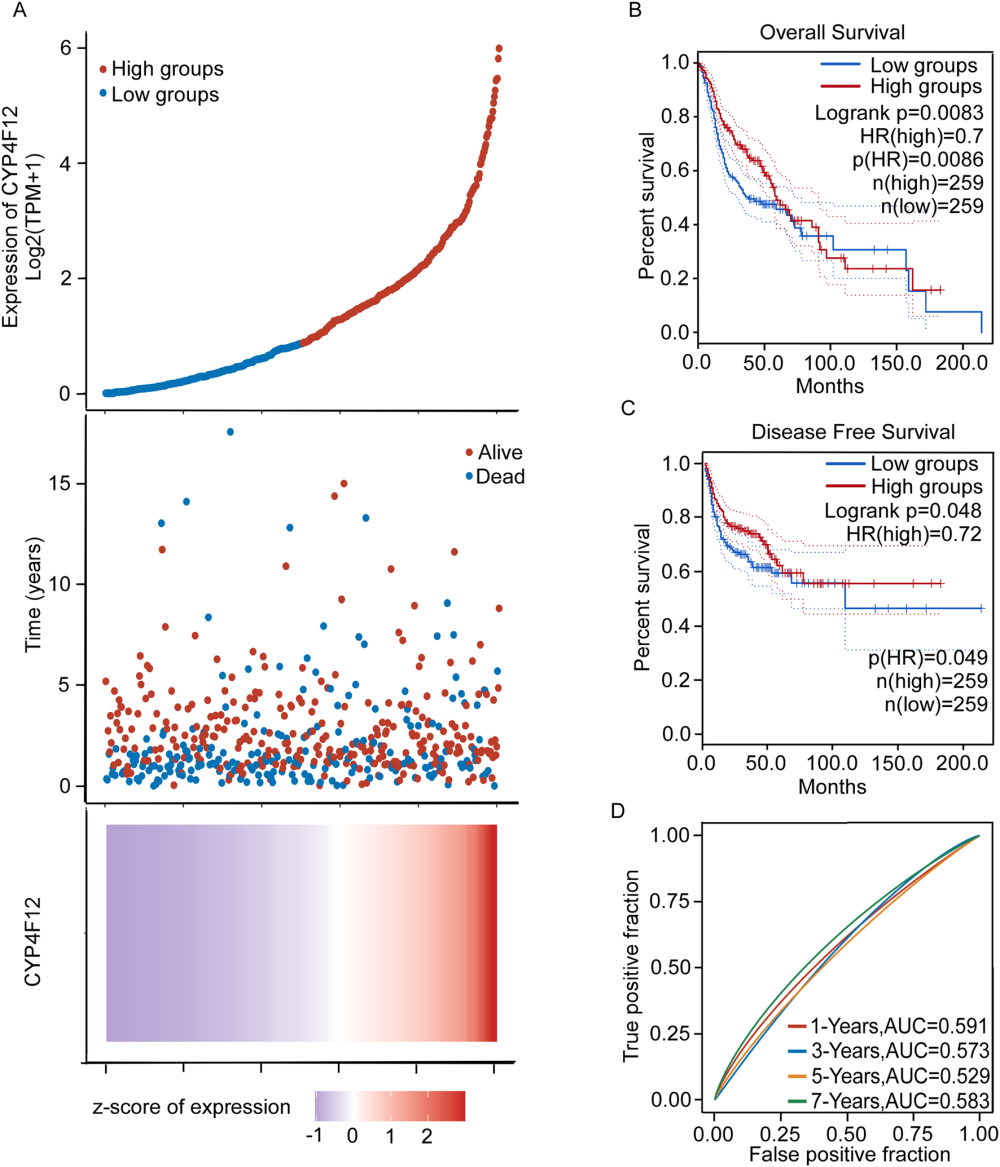
Prognostic value of CYP4F12 in HNSC patients. Patients in the TCGA HNSC dataset were grouped using the median expression of CYP4F12 as a threshold. (**A**) The expression of CYP4F12, survival time and survival status of HNSC patients in the TCGA dataset. The top of which represents the scatter plot of the gene expression from low to high, while different colors represent different groups. The middle represents the survival time corresponding to the gene expression of different samples and survival status scatter plot distribution; the bottom figure represents the expression heat map of CYP4F12. (**B** and **C**) GEPIA analyses of OS (**B**) and DFS (**C**) in HNSC patients based on CYP4F12 expression (high group vs low group). (**D**) ROC curves of CYP4F12 in predicting patient survival. Higher values of AUC represent higher predictive power.

To investigate whether CYP4F12 expression was associated with the prognosis of HNSC patients, we grouped patients in the TCGA HNSC dataset based on median expression of CYP4F12 as the critical value. CYP4F12 expression profile and patient survival status distribution was shown in Fig. 5A. GEPIA results showed that patients with higher CYP4F12 expression had better OS and DFS than those with lower CYP4F12 expression (Fig. 5B, C). And AUCs of 1-year, 3-year, 5-year and 7-year were greater than 0.5 for OS, indicating that CYP4F12 has a low prognostic predictor potential in HNSC patients (Fig. 5D).

### The relationship between CYP4F12 and clinicopathological features of patients with HNSC

Next, we summarized the clinical and molecular characteristics of HNSC patients from the TCGA HNSC data-set based on CYP4F12 expression levels. As shown in Table 2, the age of CYP4F12 high-expression group was lower than that of the low-expression group, the infection rate of HPV in the high-expression group was significantly higher than that in low-expression group, and the perineural invasion was also less frequent. In addition, there were no significant differences between CYP4F12 high group and CYP4F12 low group (*P* > 0.05) in terms of gender, pathological stage, tumor grade, smoking, alcohol history, radiationtherapy, lymphovascular invasion, and new tumor event type. These results suggest that the expression level of CYP4F12 may be associated with certain phenotypes of HNSC.

**Table 2:** Correlation of CYP4F12 expression with clinicopathological characteristics in the TCGA HNSC dataset.

Patient characteristics	CYP4F12 expression		*p* value
	High (n = 247)	Low (n = 248)	
Sex, male/female	181/66	182/66	0.978
Median age, years (range)	60(19–85)	62(24–90)	0.018
Pathologic stage			
Stage I	14	11	0.538
Stage II	44	37	0.396
Stage III	39	52	0.132
Stage IVA	148	143	0.652
Stage IVB	5	8	0.399
Stage IVC	2	1	0.563
Tumor grade			
G1	36	26	0.159
G2	148	153	0.771
G3	55	64	0.384
G4	2		0.154
GX	8	9	0.825
HPV status by p16&ISH testing (yes/no)	24/38	8/41	0.01
Smoking (yes/no)	192/53	189/60	0.515
Alcohol history (yes/no)	166/74	162/82	0.514
Radiation therapy (yes/no)	64/32	54/28	0.909
Lymphovascular invasion present (yes/no)	52/103	67/116	0.557
Perineural invasion present (yes/no)	60/102	104/84	< 0.001
New tumor event type			
Metastasis	9	10	0.968
Primary	6	3	0.222
Recurrence	17	22	0.420

### Correlation of CYP4F12 Expression with Immune in HNSC

**Figure 6:**
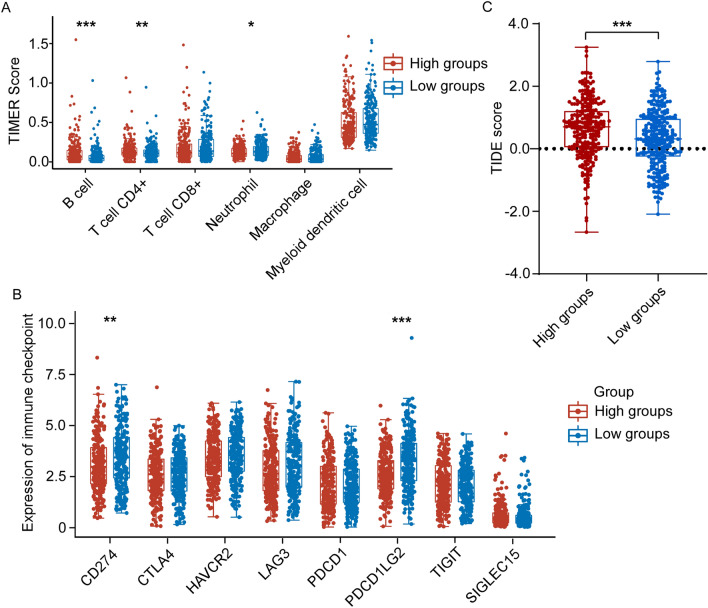
The relationship between immune scores and CYP4F12 expression level in HNSC. Patients in the TCGA HNSC dataset were grouped using the median expression of CYP4F12 as a threshold. (**A**) The expression distribution of immune score in CYP4F12 high expression group and low expression group. The abscissa represents the type of immune cell infiltration, and the ordinate represents the distribution of the immune infiltration score in different groups. (**B**) The expression distribution of immune checkpoints gene in CYP4F12 high expression group and low expression group. Horizontal coordinates indicate different groups of samples, vertical coordinates indicate the distribution of immune checkpoint gene expression, and different colors indicate different groups. (**C**) Distribution of immune response scores among different groups. Asterisks (*) stand for significance levels, *p < 0.05, **p < 0.01, ***p < 0.001.

As shown in Fig. 4 and Table 1, CYP4F12 is significantly correlated with the level of immune cells infiltration in HNSC, suggesting that CYP4F12 may affect the prognosis of HNSC patients by regulating the level of immune cell infiltration. Therefore, we analyzed the relationship between CYP4F12 expression and infiltration levels of various immune cell in HNSC. The results showed that the immune score of B cells and CD4+ T cells was significantly higher in the high expression group of CYP4F12 than that in the low expression group (Fig. 6A). We then examined the immune checkpoint and found that low expression of CYP4F12 was accompanied by high expression of CD274 and PDCD1LG2 (Fig. 6B).

Finally, we used TIDE algorithm to predict the response of TCGA HNSC samples to predictive immune checkpoint inhibitors based on expression profile data. The TIDE algorithm utilizes a group of gene expression markers to assess two different mechanisms of tumor immune escape (cytotoxic T lymphocyte (CTL) dysfunction and CTL rejection caused by immunosuppressive factors). As shown in Fig. 6C, the low expression group of CYP4F12 had a high TIDE score, indicating that the efficacy of ICB was low and the survival time after ICB treatment was shorter. In summary, low expression of CYP4F12 is associated with immunosuppression and immune escape.

### Correlation analysis between CYP4F12 expression and pathways

Next, we performed the analysis of differentially expressed genes in CYP4F12 high and low expression groups and found 102 up-regulated genes and 21 down-regulated genes (Figure S1A, B). GO and KEGG analyses were then performed on these differentially expressed genes. KEGG’s result (Figure S1C, D) showed that they may be involved in xenobiotic metabolism by cytochrome P450, the estrogen signaling pathway, and the age-rage signaling pathway in diabetes complications; while the GO analysis showed that (Figure S1E, F) they may be related to skin development, epidermis development, organization of extracellular structures and organization of extracellular matrix.

**Figure 7:**
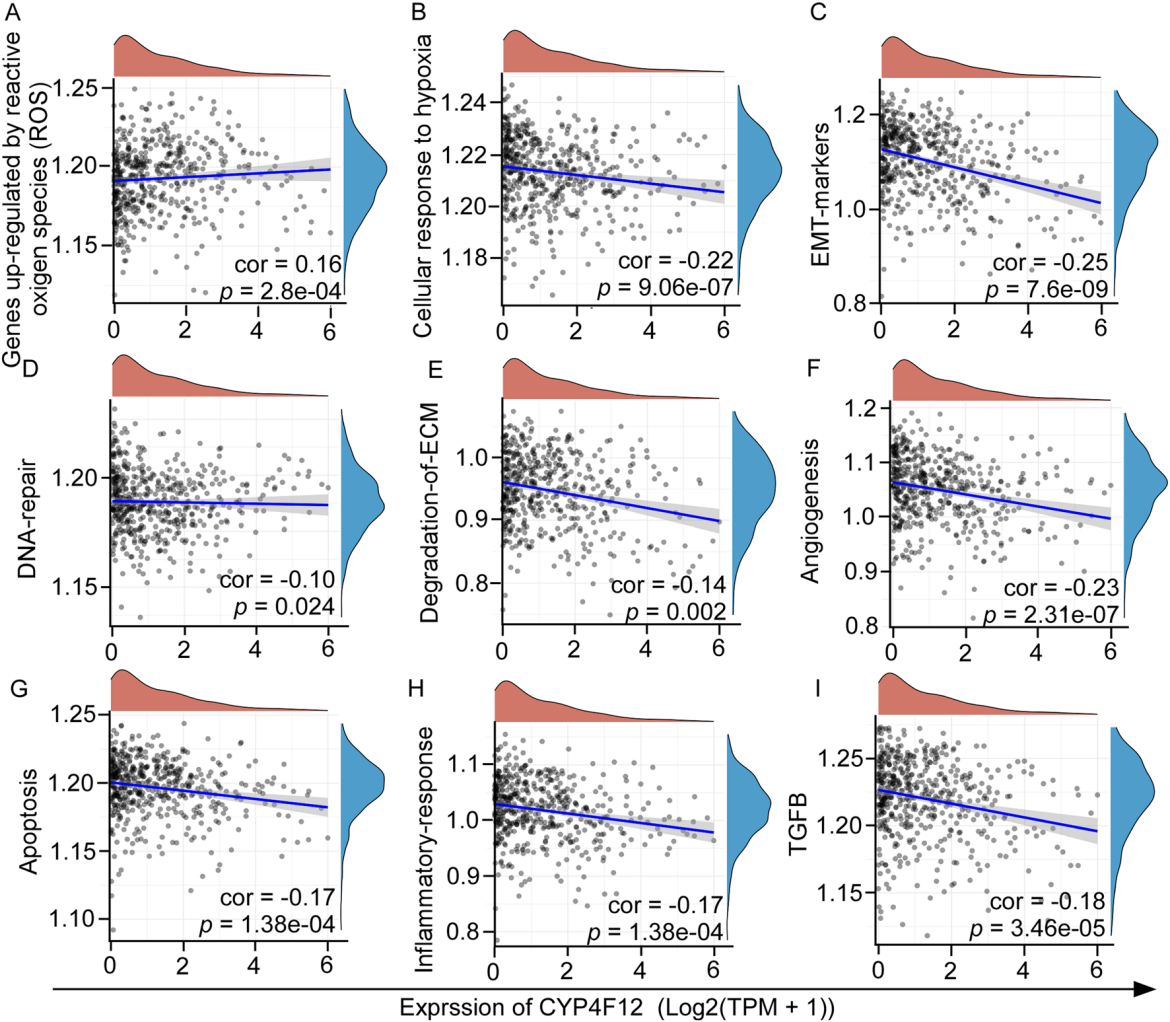
The correlations between CYP4F12 expression and pathway scores. Spearman correlation analysis between CYP4F12 expression and reactive oxygen species (ROS) (**A**), Cellular response to hypoxia (**B**), EMT markers (**C**), DNA repair (**D**), Degradation of ECM (**E**), Angiogenesis (**F**), Apoptosis (**G**), Inflammatory response (**H**) and TGFB (**G**) enriched pathway scores. The horizontal coordinates indicate the distribution of CYP4F12 expression, and the vertical coordinates indicate the distribution of each pathway score. The density curve on the right side shows the distribution trend of pathway scores, and the density curve on the top side shows the distribution trend of CYP4F12 expression. The values at the bottom right indicate the correlation coefficients and their *p*-values.

Next, according to a previously described method and the ‘ssgsea’ algorithm, we collected genes that are potentially involved in relevant pathways. Then we calculated the enrichment fractions of CYP4F12 and key pathways of tumor development to obtain the correlation between genes and pathways. The results showed that there was a positive correlation between the expression of CYP4F12 and reactive oxygen species (Fig. 7A); a negative correlation between the expression of CYP4F12 and the cellular response to hypoxia (Fig. 7B), EMT markers (Fig. 7C), DNA repair (Fig. 7D), ECM degradation of ECM (Fig. 7E), angiogenesis (Fig. 7F), apoptosis (Fig. 7G), inflammatory response (Fig. 7H), and TGFB pathway (Fig. 7I) in HNSC. Based on the above pathway analyses, we assume that CYP4F12 may play a key role in migration and apoptosis in tumor cells.

### CYP4F12 inhibits HNSC cell migration

**Figure 8:**
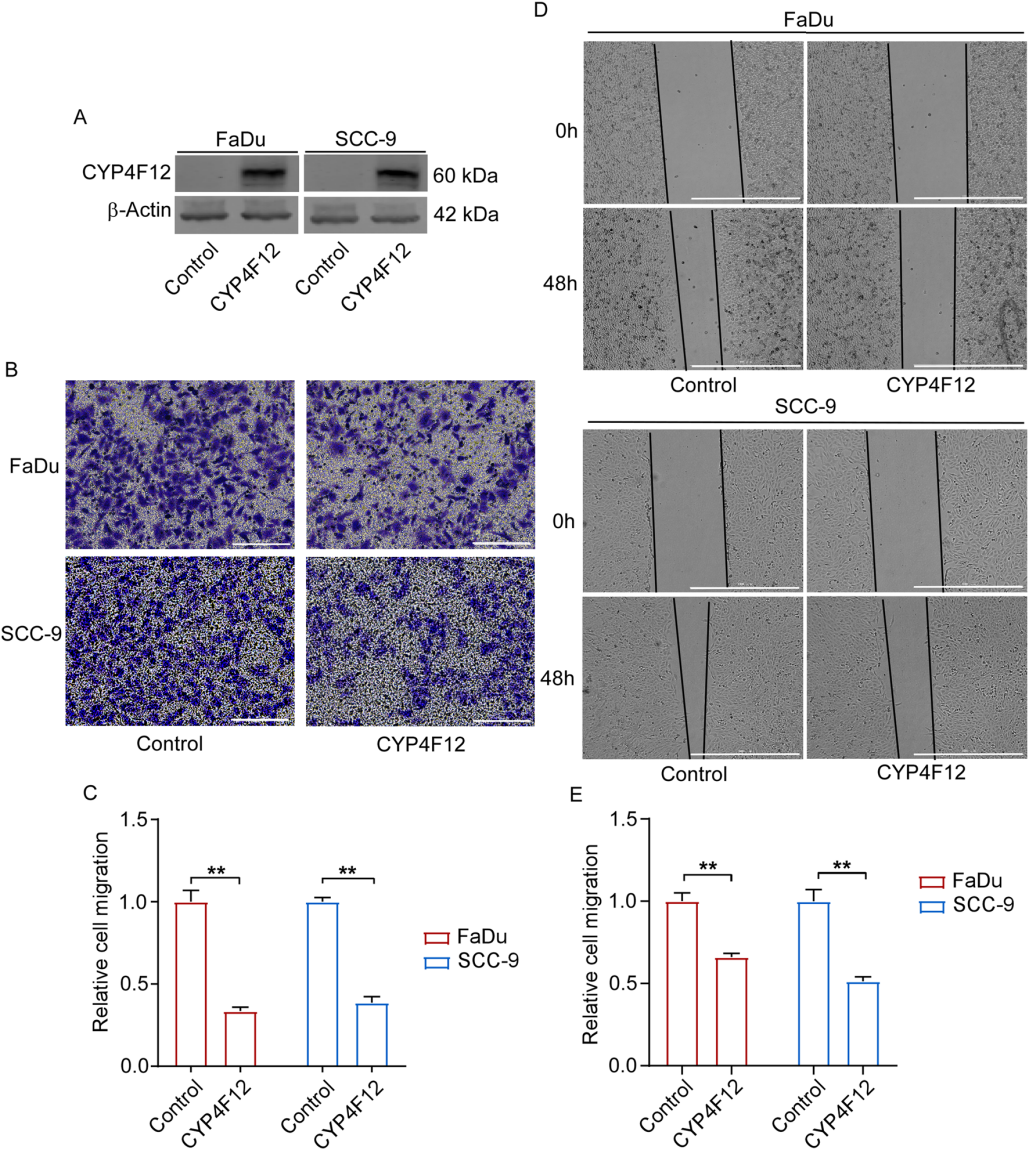
CYP4F12 inhibited HNSC cell migration. (**A**) The expression level of exogenous CYP4F12 in FaDu and SCC-9 cells was detected after transfected with CYP4F12. The original Western blot images are presented in Supplementary Figure S5. (**B**) Transwell migration assay was applied in FaDu and SCC-9 cells transfected with either control or CYP4F12 (Scale bars = 200 μm). (**C**) Quantitative analysis of transwell migration assay (n = 3, **p < 0.01). (**D**) Wound healing assay was applied in FaDu and SCC-9 cells transfected with either control or CYP4F12 (Scale bars = 1000 μm). (**E**) Quantitative analysis of wound healing assay (n = 3, **p < 0.01).

To further investigate the cellular function of CYP4F12 in HNSC, we selected two HNSC cell lines (FaDu and SCC-9) with almost undetectable endogenous expression of CYP4F12 for the following experiments. FaDu and SCC-9 cells were transfected with CYP4F12 over-expression plasmid and Western blot was used to detect the expression level of CYP4F12 after transfection. It could be seen that the expression level of CYP4F12 in FaDu and SCC-9 cells increased significantly after transfection (Fig. 8A).

We then investigated the cellular functions that CYP4F12 might regulate. CCK-8 assay was used to detect cell proliferation and flow cytometry was used to detect cell cycle and cell apoptosis. The results showed that CYP4F12 had no significant effect on cell proliferation (Figure S2A, B), cell apoptosis (Figure S2C, D) and cell cycle (Figure S3A–D).

Next, we used transwell migration assay and wound healing assay to study the potential effects of CYP4F12 on the migration ability of FaDu and SCC-9 cells. In the transwell assay, the migrated cells in the experimental groups were significantly decreased compared with the control group (Fig. 8B, C). In wound healing assay, the migration area of FaDu and SCC-9 cells was also significantly reduced in experimental groups (Fig. 8D, E). Next, we examined the knockdown of CYP4F12 expression (Figure S4A) and tested its role in migration in FaDu and SCC-9 cells. The results showed that knockdown of CYP4F12 expression did not significantly alter the migratory function of the cells (Figure S4B–F), which may be related to the low background expression of CYP4F12 in FaDu and SCC-9.The results above show that, CYP4F12 can inhibit migration of FaDu and SCC-9 cells.

### CYP4F12 improves cell adhesion

**Figure 9:**
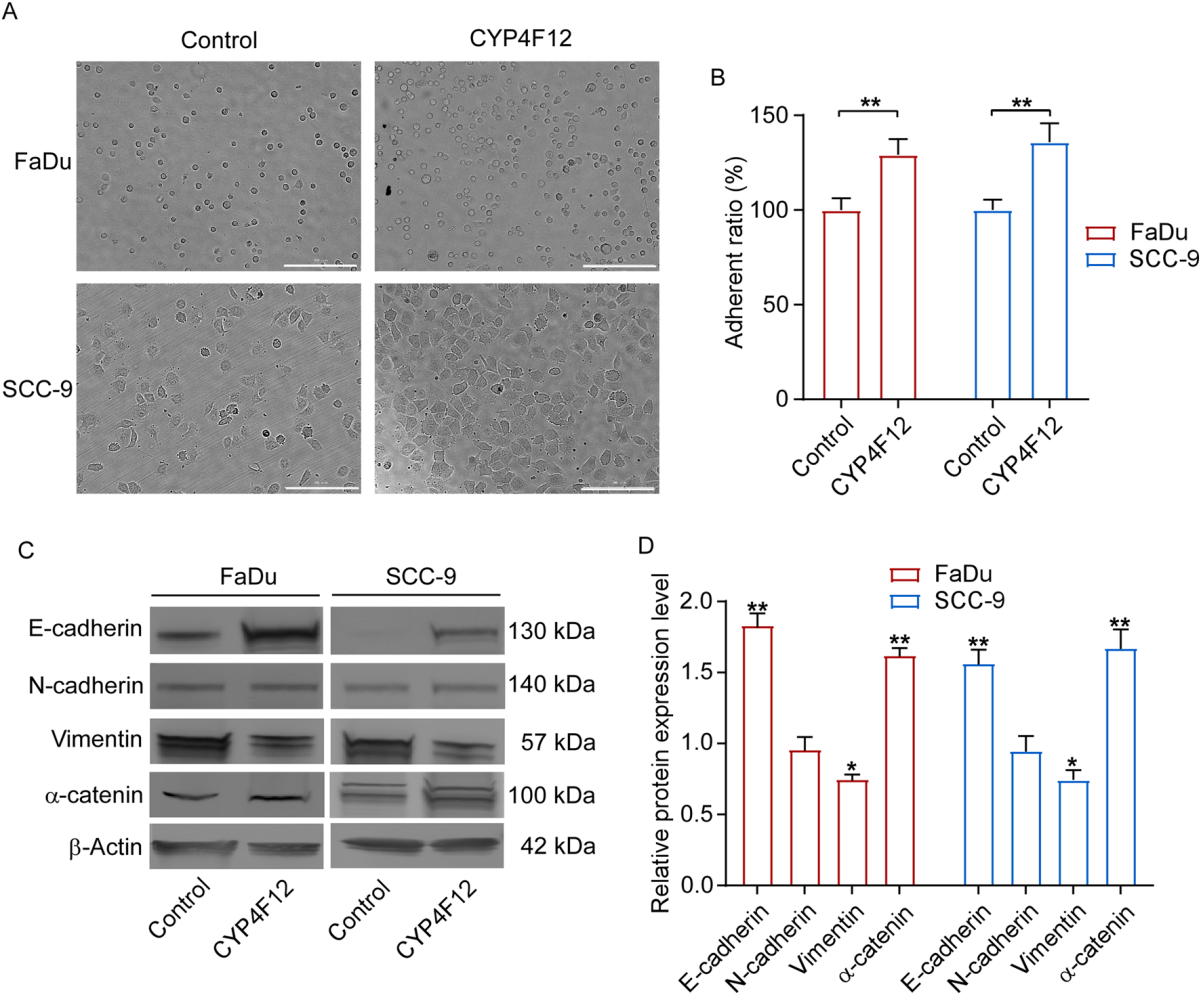
CYP4F12 enhanced cell adhesion and inhibited EMT process in HNSC cell. (**A**) CYP4F12 can increase the adhesion of FaDu and SCC-9 cells to the matrix (Scale bars = 200 μm). (**B**) The number of FaDu and SCC-9 cells in 96-well plates were detected using CCK-8 reagent. (**C**) The expressions of E-cadherin, N-cadherin, Vimentin and α-catenin were detected in FaDu and SCC-9 cells transfected with control or CYP4F12 by western blot. The original Western blot images are presented in Supplementary Figure S5. (**D**) Quantitative analysis of western blot. Values are means of triplicates (*p < 0.05, ** p < 0.01).

Since cell–matrix adhesion plays a significant role in tumor cell migration and invasive potential^34^, we performed a cell–matrix adhesion assay to evaluate whether CYP4F12 affects cell adhesion. Compared to the control, FaDu and SCC-9 cells transfected with CYP4F12 plasmid were more difficult to wash off in 96-well plate, and there were more cells left in the plate (Fig. 9A). The adhesion capacity of FaDu and SCC-9 cells to the matrix was improved in the experimental groups (Fig. 9B). Therefore, CYP4F12 can enhance the adhesion ability of FaDu and SCC-9 cells.

### CYP4F12 regulate cell migration and adhesion through EMT pathway

Since cell migration and cell–matrix adhesion are closely related to the EMT process^35,36^, we next examined whether over-expression of CYP4F12 affects the expression of EMT-related proteins in FaDu and SCC-9 cells. The result showed that compared with the control group, E-cadherin and α-catenin expression were increased in FaDu and SCC-9 cells transfected with CYP4F12, while the mesenchymal marker vimentin was down-regulated (*P* < 0.05) (Fig. 9C, D). However, N-cadherin expression was almost unaffected (Fig. 9C, D). These results suggest that CYP4F12 may affect cell migration and adhesion by regulating E-cadherin, α-catenin and vimentin.

## Discussion

Worldwide, the incidence of HNSC is rising and has the potential to overtake cervical cancer^37^. Despite advances in diagnosis and treatment in recent years, most HNSC patients, especially those with advanced disease at diagnosis, have a poor prognosis and low 5-year survival rates^38,39^. And only a small number of HNSC patients respond fully or partially to targeted drugs and ICI therapies^40,41^. Although a variety of biomarkers have been detected in HNSC in recent years, well-validated prognostic markers have not been found. This highlighted the importance of finding a biomarker for the diagnosis and therapy of HNSC.

CYP4F12 is widely expressed in human tissues and is significantly reduced in a variety of human tumors. However, the expression pattern and molecular mechanism of CYP4F12 in HNSC remain unclear. In this study, we found that the mRNA level of CYP4F12 was decreased in tumor tissues. We then verified the expression of CYP4F12 in the GEO database and patient specimens collected by our laboratory, the results also showed that CYP4F12 was low expressed in tumor tissues. Then we investigated the relationship between CYP4F12 expression and clinical characteristics of HNSC, and found that CYP4F12 expression was correlated with patient age, gender, ethnicity, HPV infection, TP53 mutation and tumor grade. In particular, with regard to tumor grading, we found that CYP4F12 expression gradually decreased in grades 1–3 and reached the highest in grade 4. With the increase of tumor grade, the infiltration and spreading rate of cancer cells increased, and the expression of CYP4F12 should be gradually decreased, but there is an abnormally high expression in grade 4 tumor cells. We think it may be related to the following two points: firstly, the number of cases of grade 4 tumors is too small compared with these in other grades; secondly, grade 4 tumors are hypodifferentiated tumor cells with distinctly different characteristics and gene expression than highly differentiated tumor cells in which CYP4F12 may have different functions.

Preclinical data show that HNSC is a severe immunosuppressive disease, and the occurrence and development of head and neck squamous cell carcinoma is accompanied by abnormal secretion of pro-inflammatory cytokines and dysfunction of immune effector cells^42–44^. Therefore, we next tested the correlation between the expression of CYP4F12 and immunity, and found that the expression of CYP4F12 was positively correlated with the expression of B cells and CD4+ T cells in HNSC, and was positively correlated with the immune markers of B cells, CD8+ T Cells, CD4+ T Cells and M1 macrophage. The above results show that CYP4F12 is positively correlated with immunity in HNSC, and affects the expression of some immune markers.

Next, we grouped TCGA patients according to the expression of CYP4F12, and studied the differences of different CYP4F12 expression levels in clinical phenotype, immunity and pathways. We found that the low CYP4F12 expression group had worse OS and DFS, and had higher average age, lower HPV positive rate and higher perineural invasion present. And we also found that low CYP4F12 expression group had lower immune activity, stronger immunosuppression, worse ICB efficacy and shorter survival after ICB treatment. It could be seen that the low expression of CYP4F12 affects part of the clinical phenotypes of HNSC patients and inhibits immune activity while improving immunosuppression.

Then we analyzed the expression differences between the two groups with different levels of CYP4F12, and obtained 102 up-regulated genes and 21 down-regulated genes. We performed GO and KEGG analysis on the differentially expressed genes, KEGG results indicated that they might be involved in xenobiotic metabolism of cytochrome P450, estrogen signaling pathway and age-rage signaling pathway in diabetic complications; GO analysis indicated that they might be involved in skin development, epidermal development, extracellular structure organization and extracellular stromal tissue.

Afterward, according to literature we obtained the correlation between CYP4F12 and some key pathways in tumors by calculating the correlation between gene expression and pathway score^45^, and finally we found that cellular response to hypoxia, EMT markers, DNA repair, degradation of ECM, angiogenesis, apoptosis, inflammatory response, TGFB pathway were significantly negatively correlated with the expression of CYP4F12.

Finally, we verified the results of bioinformatics analysis. We analyzed the proliferation, apoptosis, cell cycle changes and migration of FaDu and SCC-9 cells after high expression of CYP4F12. The results showed that high expression of CYP4F12 had no significant effect on the proliferation, apoptosis and cell cycle changes of HNSC cells, but high expression of CYP4F12 could inhibit the migration of HNSC cells and enhance cell adhesion.

EMT is a developmental process that plays a key role in embryogenesis, wound healing and organ fibrosis^46,47^. EMT also promotes cancer progression by promoting the loss of intercellular adhesion, resulting in increased cell migration and invasive capacity^48^. EMT hallmarks include loss of E-cadherin expression and concomitant increase in mesenchymal markers such as N-cadherin, vimentin^49^. Therefore, we next detected the expression of several EMT markers, and Western blotting results showed that high expression of CYP4F12 inhibited EMT progression in FaDu and SCC-9 cells by increasing the expression of E-cadherin, α-catenin and decreasing the expression of vimentin.

Studies have shown that many oncogenes, just as c-Myc, FAK and Ras-MAPK, have been identified as regulators of multiple signaling pathways during tumor progression, including intercellular adhesion, migration, proliferation and chemokine transcription. At the same time they are involved in tumor immune regulation in different cancer models, affecting the tumor immune microenvironment^50–53^. In our study, CYP4F12 played a role in the immune infiltration of HNSC and high expression of CYP4F12 in vitro inhibited the migration of HNSC cell lines. Therefore, CYP4F12 can regulate cell migration by directly affecting the EMT process in HNSC, and may also affect tumor cell migration by influencing the tumor microenvironment.

Although this study has identified CYP4F12 as a latent factor in the pathogenesis of HNSC, there are still several shortcomings. First, given the complex interactions between tumors and the microenvironment, we have not demonstrated in vivo that CYP4F12 has tumor suppressive effects. Second, although we have discovered the role of CYP4F12 in HNSC migration, its potential mechanism requires further in-depth studies to uncover the regulatory effects of CYP4F12 on E-cadherin, α-catenin and vimentin.

In conclusion, this study demonstrates that CYP4F12 expression affects HNSC progression and metastasis, and that its low expression is associated with a worse patient prognosis. Since CYP4F12 has been shown to inhibit tumor cell migration in vitro, we hypothesized that CYP4F12 may be a promising HNSC biomarker that could predict anticancer therapy efficacy and patient prognosis.

## Supplementary Information

Supplementary Figures.

## Data Availability

Publicly available datasets were used for analysis in this study. Data sources are described in Materials and Methods.
