# Supplementary material for: CYP4F12 is a potential biomarker and inhibits cell migration of head and neck squamous cell carcinoma via EMT pathway
Source: Sci Rep. 2023 Jul 6;13:10956. doi: 10.1038/s41598-023-37950-z (PMC10326030; doi:10.1038/s41598-023-37950-z)
Supplement: Supplementary file 1 — Supplementary Figures. [file 41598_2023_37950_MOESM1_ESM.docx]

*CYP4F12* is a potential biomarker and inhibits cell migration of head and neck squamous cell carcinoma via EMT pathway

Wenming Jia ^1^, Shuai Chen ^1^, Ran Wei ^1^, Xiaoqi Yang ^1^, Minfa Zhang ^2^, Ye Qian ^1^，Heng Liu ^1,^* and Dapeng Lei ^1,^*


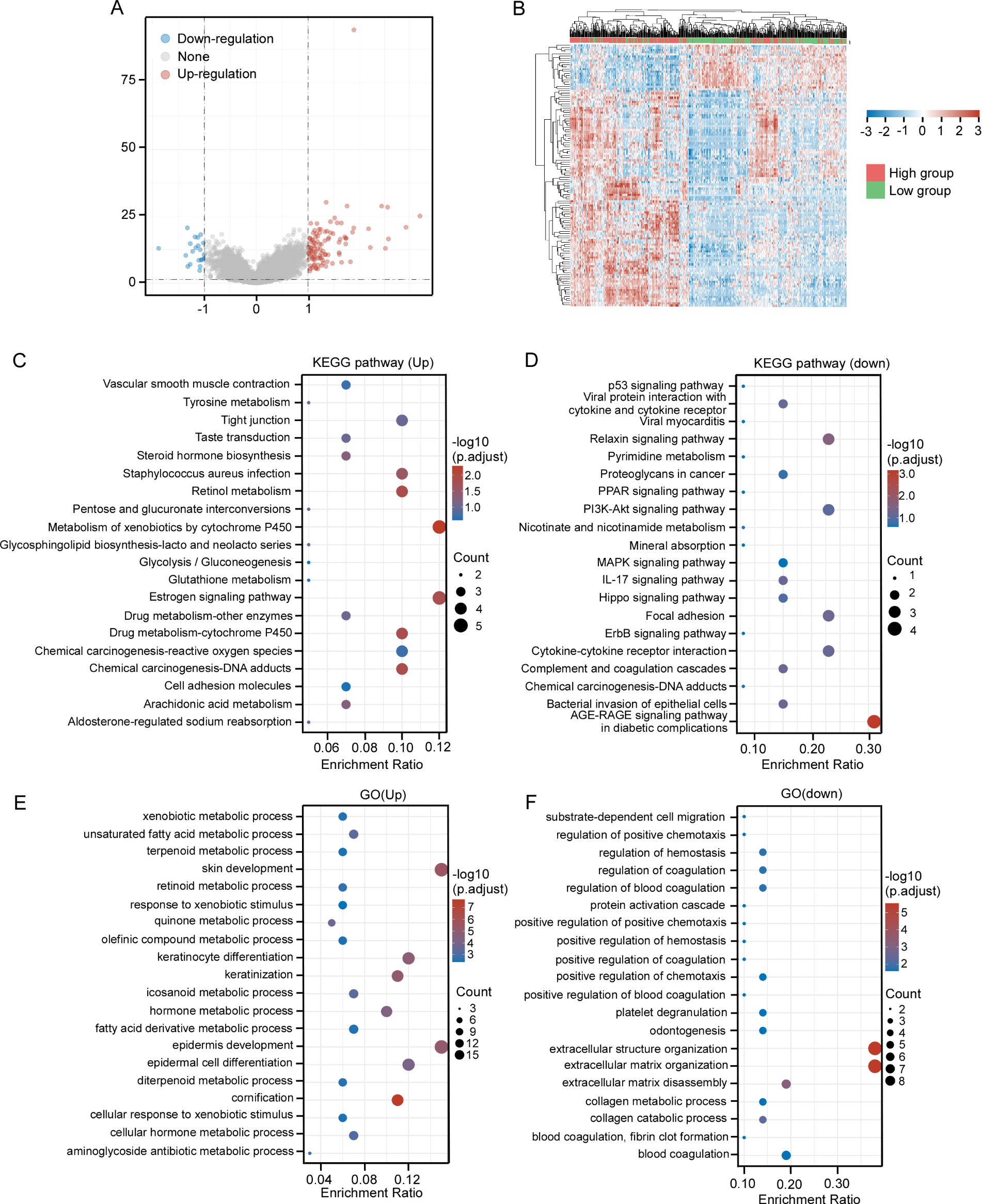


**Figure S1.** **Differentially expressed gene analysis and KEGG pathway analysis of CYP4F12 high and low expression groups.** (A) Volcano plot using fold change and corrected *p*-values. The red dots represent significantly up-regulated genes, the blue dots represent significantly down-regulated genes, and the grey dots represent those without significant changes. (B) Heat map of differently expressed genes, where colors represent expression trends in different tissues. Due to the large number of differently expressed genes, only the most significantly up-regulated genes (n=50) and down-regulated genes (n=50) are displayed here. (C) KEGG pathway enrichment results for significantly up-regulated genes; (D) KEGG pathway enrichment results for significantly down-regulated genes. (E)GO term enrichment results for differentially up-regulated genes; (F) GO term enrichment results for differentially down-regulated genes. Different colors represent the significance of the differential enrichment results. The size of the circle represents the number of enriched genes.


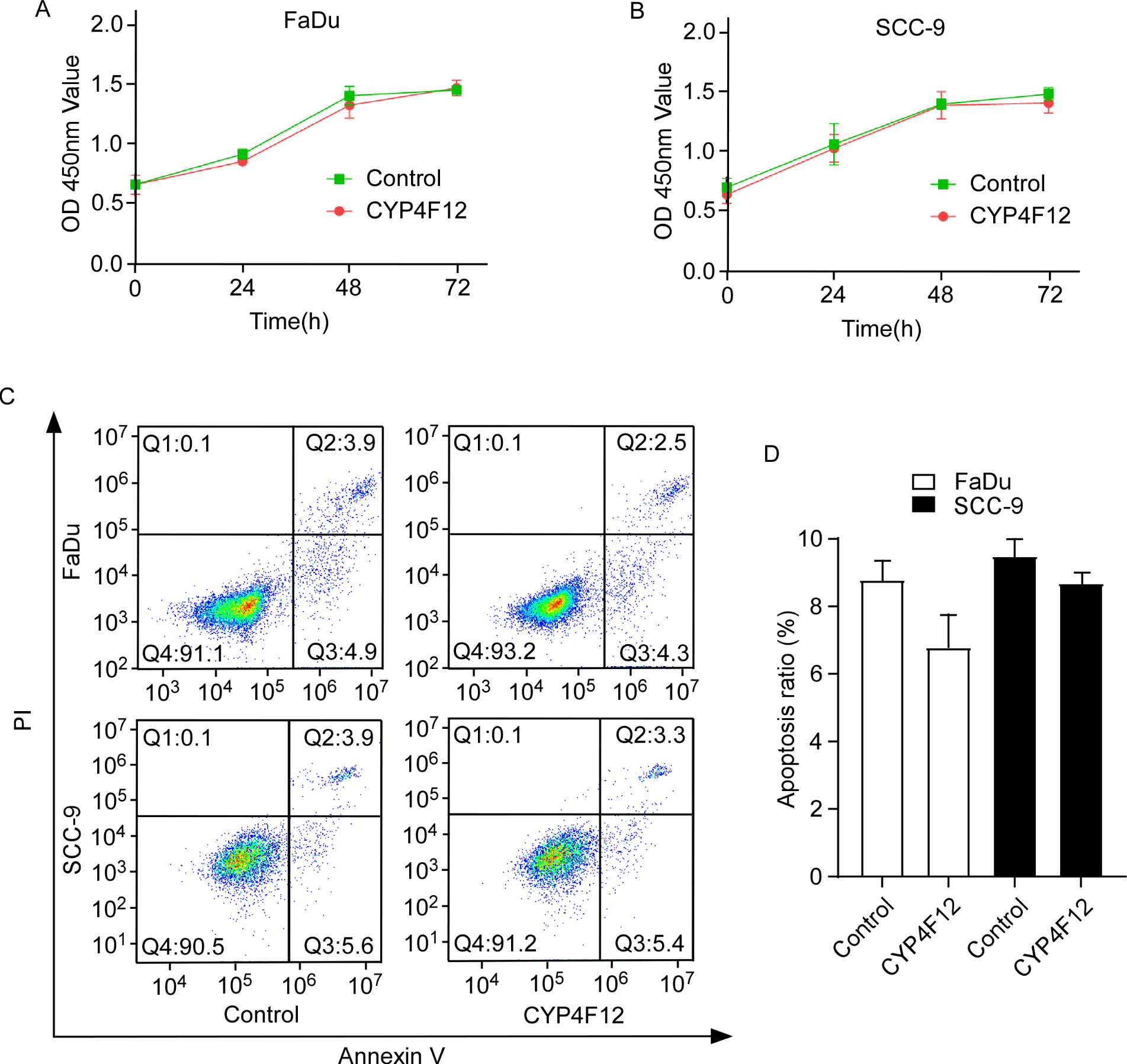


**Figure S2. Effects of CYP4F12 on proliferation and cell apoptosis in vitro.** (A, B) The effect of CYP4F12 on cell proliferation was examined in FaDu and SCC-9 cell lines. (C) The effect of CYP4F12 on apoptosis was examined in FaDu and SCC-9 cell lines. (D) Quantitative analysis of cell apoptosis assay.


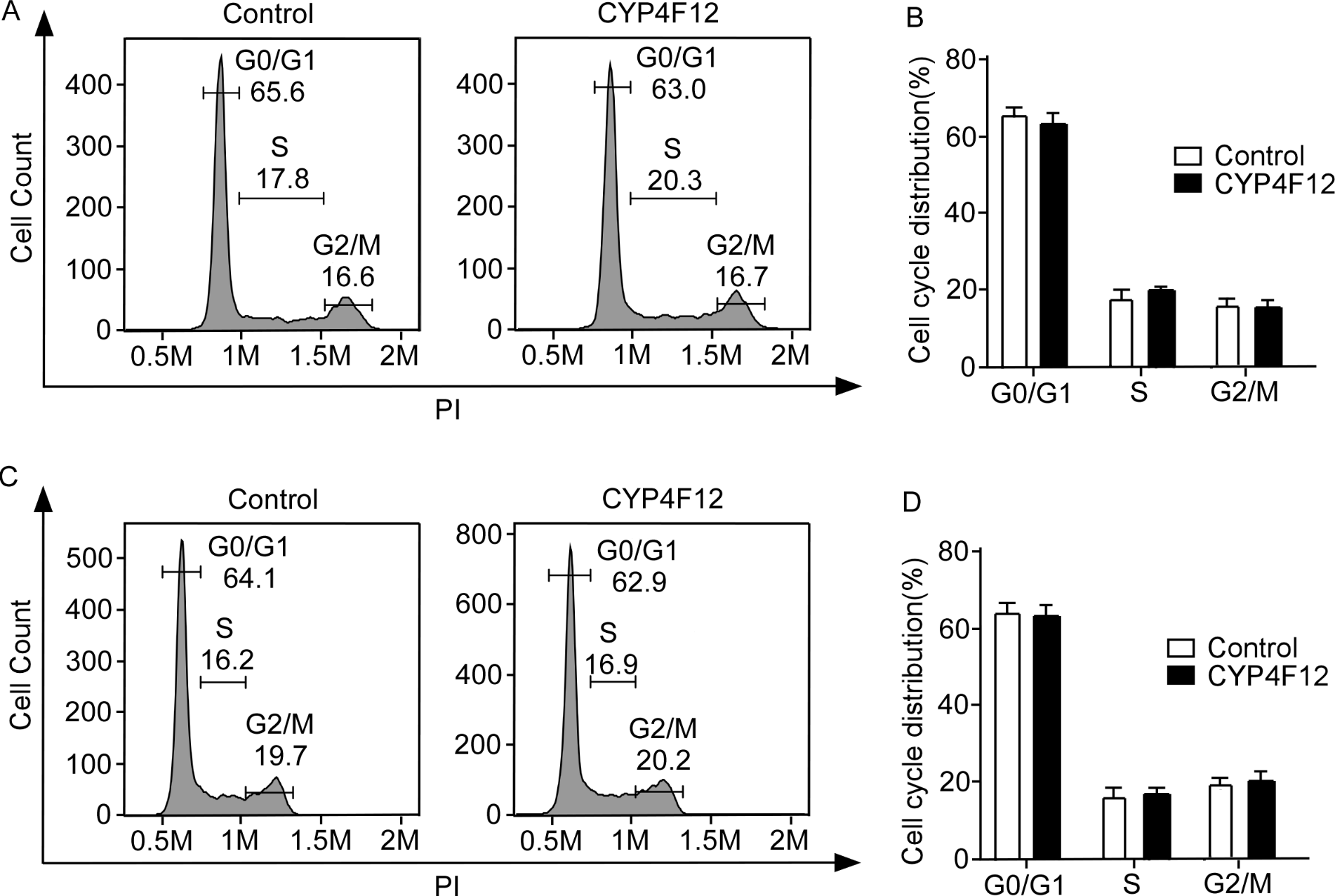


**Figure S3.** **Effects of CYP4F12 on cell cycle.** (A) The effect of CYP4F12 on cell cycle was examined in FaDu cell line. (B) Quantitative analysis of cell cycle assay. (C) The effect of CYP4F12 on cell cycle was examined in SCC-9 cell line. (D) Quantitative analysis of cell cycle assay.


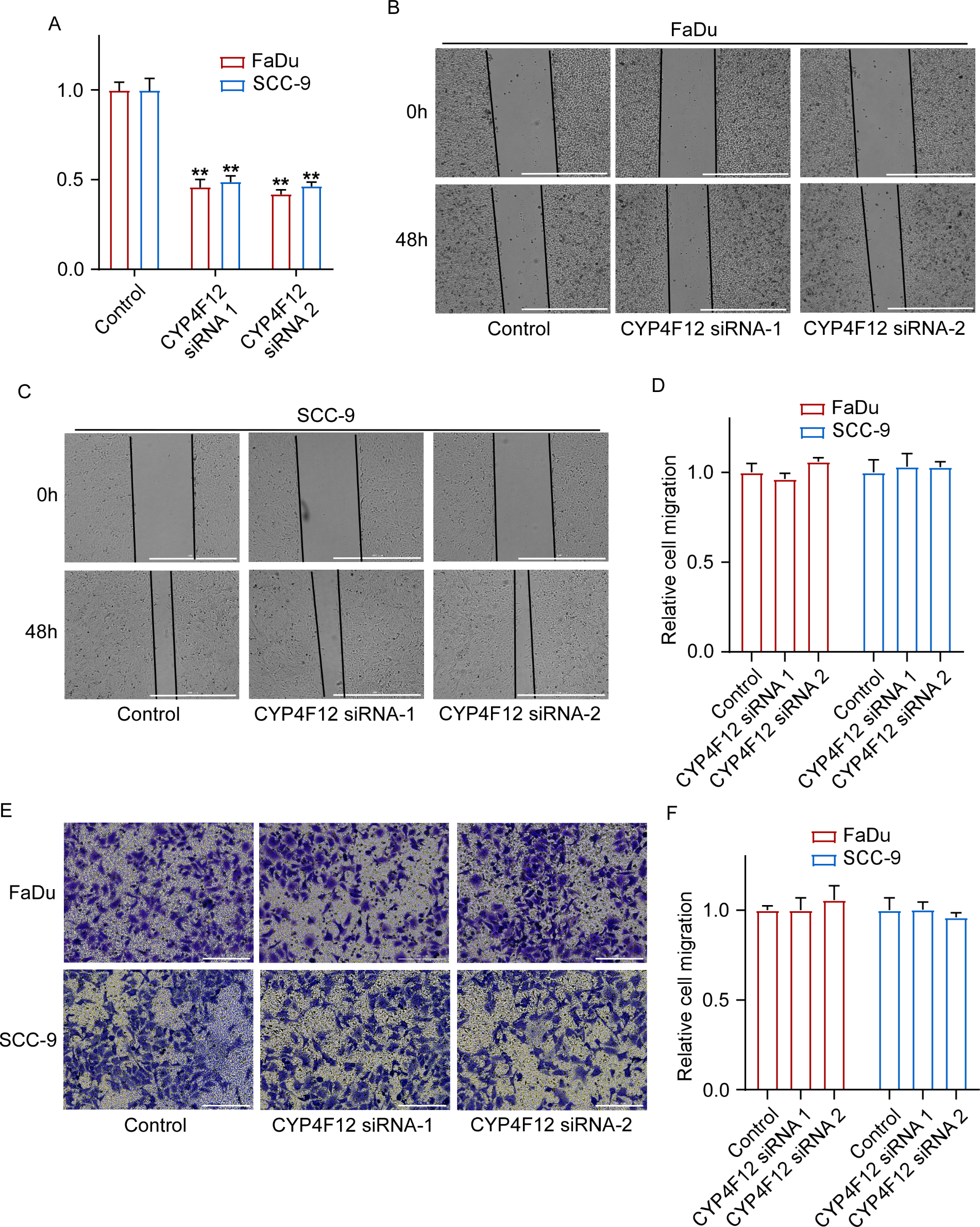


**Figure S4. Effect of knockdown of CYP4F12 expression on migration of FaDu and SCC-9 cells.** (A) The expression of CYP4F12 in FaDu and SCC-9 after transfection with CYP4F12 siRNA was detected by qRT-PCR (n = 3, **p < 0.01). (B-C) Wound healing assay was applied in FaDu(B) and SCC-9(C) cells transfected with either control or CYP4F12 siRNA (Scale bars = 1000 μm). (D) Quantitative analysis of wound healing assay. (E) Transwell migration assay was applied in FaDu and SCC-9 cells transfected with either control or CYP4F12 siRNA (Scale bars = 200 μm). (C) Quantitative analysis of transwell migration assay.

**
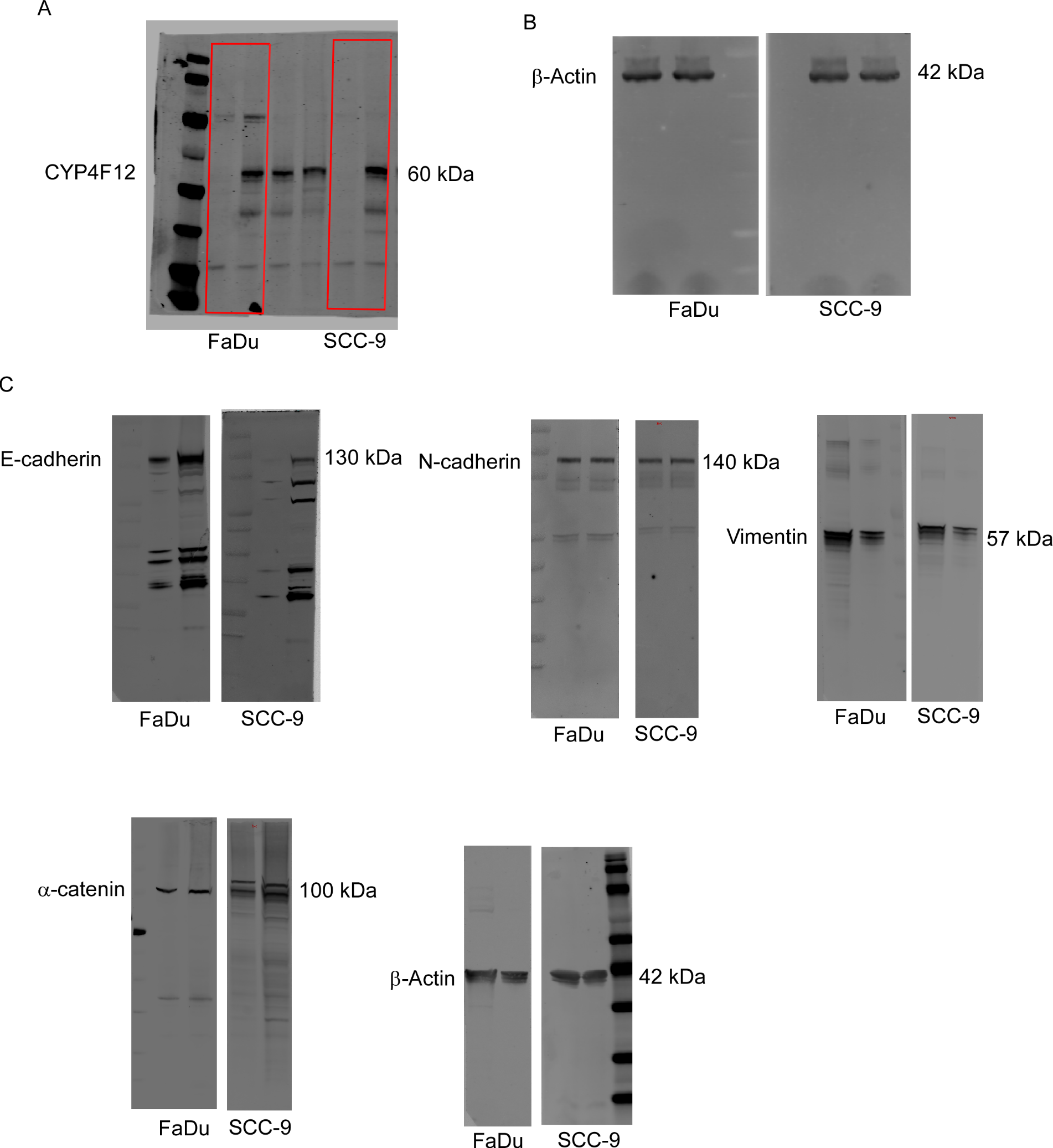
**

**Figure S5. Western blots original images.** (A) The Western blot images corresponding to Figure 8A. (B) The Western blot images corresponding to Figure 8A. (C) The Western blot images corresponding to Figure 9C.
